# Supplementary material for: HIVprotI: an integrated web based platform for prediction and design of HIV proteins inhibitors
Source: J Cheminform. 2018 Mar 9;10:12. doi: 10.1186/s13321-018-0266-y (PMC5845081; doi:10.1186/s13321-018-0266-y)
Supplement: Supplementary file 2 — Additional file 2. Source code of HIVProtI web server. [file 13321_2018_266_MOESM2_ESM.zip › HIVprotI_Source-code/hivprotI/weka-3-6-6/doc/weka/gui/streams/InstanceJoiner.html]

InstanceJoiner


---


|  |  |  |
| --- | --- | --- |
| **Overview** \| **Package** \| **Class** \| **Tree** \| **Deprecated** \| **Index** \| **Help** \| **Weka's home** | |  |
| **PREV CLASS**   **NEXT CLASS** | **FRAMES**    **NO FRAMES**     **All Classes** |  |
| SUMMARY: NESTED \| FIELD \| CONSTR \| METHOD | DETAIL: FIELD \| CONSTR \| METHOD |  |


---


## weka.gui.streams Class InstanceJoiner

```
java.lang.Object
  weka.gui.streams.InstanceJoiner
```

**All Implemented Interfaces:**: java.io.Serializable, java.util.EventListener, InstanceProducer, SerialInstanceListener

---

``` public class InstanceJoiner extends java.lang.Object implements java.io.Serializable, InstanceProducer, SerialInstanceListener ```

A bean that joins two streams of instances into one.

**Version:**
:   $Revision: 7059 $

**Author:**
:   Len Trigg (trigg@cs.waikato.ac.nz)

**See Also:**: Serialized Form

---

**Constructor Summary**

`InstanceJoiner()`
  
          Setup the initial states of the member variables


| **Method Summary** | |
| --- | --- |
| `void` | `addInstanceListener(InstanceListener ipl)` |
| `void` | `batchFinished()`             Signify that this batch of input to the filter is finished. |
| `boolean` | `getDebug()` |
| `boolean` | `input(Instance instance)` |
| `boolean` | `inputFormat(Instances instanceInfo)`             Sets the format of the input instances. |
| `void` | `instanceProduced(InstanceEvent e)` |
| `Instances` | `outputFormat()`             Gets the format of the output instances. |
| `Instance` | `outputPeek()`             Output an instance after filtering but do not remove from the output queue. |
| `void` | `removeInstanceListener(InstanceListener ipl)` |
| `void` | `secondInstanceProduced(InstanceEvent e)` |
| `void` | `setDebug(boolean debug)` |

**Methods inherited from class java.lang.Object**

`equals, getClass, hashCode, notify, notifyAll, toString, wait, wait, wait`

**Constructor Detail**

### InstanceJoiner

```
public InstanceJoiner()
```

:   Setup the initial states of the member variables


**Method Detail**

### inputFormat

```
public boolean inputFormat(Instances instanceInfo)
```

:   Sets the format of the input instances. If the filter is able to determine
    the output format before seeing any input instances, it does so here. This
    default implementation assumes the output format is determined when
    batchFinished() is called.

    :   **Parameters:**: `instanceInfo` - an Instances object containing the input instance structure (any instances contained in the object are ignored - only the structure is required). **Returns:**: true if the outputFormat may be collected immediately

---


### outputFormat

```
public Instances outputFormat()
                       throws java.lang.Exception
```

:   Gets the format of the output instances. This should only be called
    after input() or batchFinished() has returned true.

    :   **Specified by:**: `outputFormat` in interface `InstanceProducer`
    :   **Returns:**: an Instances object containing the output instance structure only. **Throws:**: `java.lang.Exception` - if no input structure has been defined (or the output format hasn't been determined yet)

---


### input

```
public boolean input(Instance instance)
              throws java.lang.Exception
```

:   **Throws:**: `java.lang.Exception`

---


### batchFinished

```
public void batchFinished()
                   throws java.lang.Exception
```

:   Signify that this batch of input to the filter is finished. If the filter
    requires all instances prior to filtering, output() may now be called
    to retrieve the filtered instances. Any subsequent instances filtered
    should be filtered based on setting obtained from the first batch
    (unless the inputFormat has been re-assigned or new options have been
    set). This default implementation assumes all instance processing occurs
    during inputFormat() and input().

    :   **Throws:**: `java.lang.Exception` - if no input structure has been defined

---


### outputPeek

```
public Instance outputPeek()
                    throws java.lang.Exception
```

:   Output an instance after filtering but do not remove from the output
    queue.

    :   **Specified by:**: `outputPeek` in interface `InstanceProducer`
    :   **Returns:**: the instance that has most recently been filtered (or null if the queue is empty). **Throws:**: `java.lang.Exception` - if no input structure has been defined

---


### setDebug

```
public void setDebug(boolean debug)
```

---


### getDebug

```
public boolean getDebug()
```

---


### addInstanceListener

```
public void addInstanceListener(InstanceListener ipl)
```

:   **Specified by:**: `addInstanceListener` in interface `InstanceProducer`

---


### removeInstanceListener

```
public void removeInstanceListener(InstanceListener ipl)
```

:   **Specified by:**: `removeInstanceListener` in interface `InstanceProducer`

---


### instanceProduced

```
public void instanceProduced(InstanceEvent e)
```

---


### secondInstanceProduced

```
public void secondInstanceProduced(InstanceEvent e)
```

:   **Specified by:**: `secondInstanceProduced` in interface `SerialInstanceListener`


---


|  |  |  |
| --- | --- | --- |
| **Overview** \| **Package** \| **Class** \| **Tree** \| **Deprecated** \| **Index** \| **Help** \| **Weka's home** | |  |
| **PREV CLASS**   **NEXT CLASS** | **FRAMES**    **NO FRAMES**     **All Classes** |  |
| SUMMARY: NESTED \| FIELD \| CONSTR \| METHOD | DETAIL: FIELD \| CONSTR \| METHOD |  |


---
